# Results of five-year systematic screening for latent tuberculosis infection in healthcare workers in Portugal

**DOI:** 10.1186/1745-6673-5-22

**Published:** 2010-07-26

**Authors:** José Torres Costa, Rui Silva, Raul Sá, Maria João Cardoso, Albert Nienhaus

**Affiliations:** 1 Occupational Health Division, Hospital S. João, EPE - Porto, Portugal; 2 Allergy Division, Hospital S. João, EPE - Porto, Portugal; 3 Medical School, Oporto University, Porto, Portugal; 4 Clinical Pathology Division, Hospital S. João, EPE - Porto, Portugal; 5 Institute for Health Service Research in Dermatology and Nursing, University Clinics Hamburg-Eppendorf, Germany

## Abstract

**Introduction:**

The risk of tuberculosis (TB) in healthcare workers (HCWs) is related to its incidence in the general population, and increased by the specific risk as a professional group. The prevalence of latent tuberculosis infection (LTBI) in HCWs in Portugal using the tuberculin skin test (TST) and the interferon-γ release assays (IGRA) was analyzed over a five-year period.

**Methods:**

A screening programme for LTBI in HCWs was conducted, with clinical evaluations, TST, IGRA, and chest radiography. Putative risk factors for LTBI were assessed by a standardised questionnaire.

**Results:**

Between September 2005 and June 2009, 5,414 HCWs were screened. The prevalence of LTBI was 55.2% and 25.9% using a TST ≥ 10 mm or an IGRA test result (QuantiFERON-TB Gold In-Tube) INF-γ ≥0.35 IU/mL as a criterion for LTBI, respectively. In 53 HCWs active TB was diagnosed. The number of HCWs with newly detected active TB decreased from 19 in the first year to 6 in 2008. Risk assessment was poorly related to TST diameter. However, physicians (1.7%) and nurses (1.0%) had the highest rates of active TB.

**Conclusions:**

LTBI and TB burden among HCWs in Portugal is high. The screening of these professionals to identify HCWs with LTBI is essential in order to offer preventive chemotherapy to those with a high risk of future progression to disease. Systematic screening had a positive impact on the rate of active TB in HCWs either by early case detection or by increasing the awareness of HCWs and therefore the precautions taken by them.

## Introduction

With the advent of antibiotics, many infectious diseases such as tuberculosis (TB) seemed well under control [1]. This feeling of security led to an absence of investment in implementing preventive measures and of training and education for healthcare workers (HCWs) on the risk of nosocomial infections and occupational diseases [2]. The emergence of groups with epidemic TB infection, i.e. HIV/AIDS-patients, further aggravated the situation [3].

In HCWs, the risk of TB infection is increased by exposure to patients with infectious disease, insufficient use of protective equipment such as respirators, and working conditions, particularly in inadequately ventilated areas and when conducting techniques which involve exposure to contaminated aerosols [3,4]. Given this higher risk of contracting the disease by exposure to *M. tuberculosis *in the workplace, in Portugal it is considered an occupational disease [5]. The incidence of TB in HCWs is related to the incidence in the general population in that geographical area. Added to this is the specific risk as a professional group, which depends on the type of health unit, workgroup, and efficiency in the implementation of infection control measures [6-9].

According to official records, the average rate of TB reported in the general population in Portugal is 29.4/100,000, which means that, despite the reduction observed in recent years, it still has the highest incidence in the EU excluding the countries of the 2004 enlargement [10]. Despite the mandatory notification of active TB, there are no official records in Portugal of the number of affected HCWs [11].

Early diagnosis and effective treatment of patients, early recognition of possible contacts, the adoption of protective measures and the effective screening for cases of latent tuberculosis infection (LTBI) are all necessary for controlling the risk of TB in HCWs [9,12,13]. The diagnosis of recent cases of LTBI (conversion) is particularly important since the lifetime risk of progression to active disease ranges from 10 to 20% [12,14]. According to several studies, the treatment of LTBI reduces the risk of developing active TB by more than 50% [15-17], and is therefore one of the main objectives of a screening programme.

Until a few years ago, contacts were screened for conversions using the tuberculin skin test (TST) [18]. In recent years, advances in molecular biology have led to the development of new in-vitro tests that measure the levels of interferon-γ released by sensitized T lymphocytes after stimulation with antigens of *M. tuberculosis*. These interferon-γ release assays (IGRA) do not present cross-reaction with Bacillus Calmet-Guérin (BCG), nor with the majority of nontuberculous mycobacteria [19]. Several HCW studies using IGRA have been performed so far comparing TST to IGRA [20-25]. While systematic screening of HCWs for TB started in 2005 at the S. João Hospital in Porto, Portugal, IGRA testing was introduced to the screening process in 2007. The subgroup in which TST and IGRA were performed simultaneously is described in previous papers [22,23]. In this paper we describe the results of the screening programme for the whole group that was screened between 2005 and 2009. Special emphasis is placed on workplace risk factors that might account for LTBI or active TB in HCWs.

## Methods

**Figure 1:**
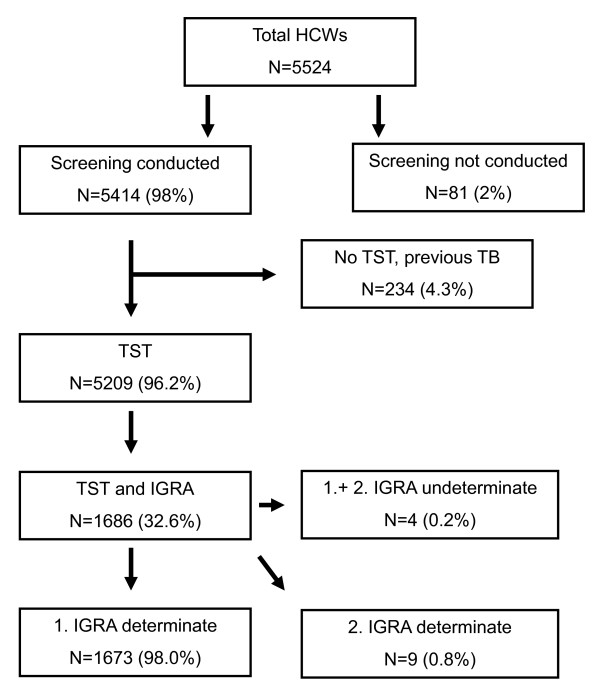
**Study population**.

The risk of *M. tuberculosis *infection was assessed in 5,524 HCWs working or training in our hospital between September 2005 and June 2009 (Figure 1). According to the latest CDC guidelines (2005) and based on the number of beds and patients diagnosed with TB each year (average of 258 patients, and 17.2 HCWs per TB patient ratio), the hospital is classified as a "medium risk" institution [9]. This screening was done on a regular annual basis, whenever new staff were employed, following occasional requests from symptomatic workers, or in cases of contact with infectious patients or materials. The risk of transmission of TB was classified as low, moderate and high, according to the CDC [9].

This screening programme consisted of: 1) a standardized interview covering gender, age, workplace, exposure situation, TB history and TB-related respiratory symptoms (cough, productive cough, haemoptysis, thoracalgia) as well as constitutional symptoms (fatigue, weight loss, fever and night sweat), 2) chest radiography (CXR) if TST or IGRA were positive or if symptomatic, 3) TST with 0.1 mL of 2 units of purified tuberculin (PPD) RT 23 SSI, unless contraindication (previous tuberculin reaction ≥15 mm, previous diagnosis of TB with appropriate treatment, severe viral infection or immunisation with vaccine with live virus less than 1 month ago, large extensive burns or eczema), and 4) since April 2007 in-vitro enzyme immunoassay (ELISA) test based on the quantification of interferon-γ, QuantiFERON-TB Gold In-Tube (IGRA). This test was carried out simultaneously with the TST [26]. A TST ≥ 10 mm was considered as positive, in accordance with Portuguese guidelines [13]. IGRA tests were carried out on the basis of the manufacture's manual and considered positive when ≥0.35 IU/mL. Undetermined IGRA results were repeated once. If screening was performed as a result of unprotected contact with infectious patients or materials, TST and IGRA were performed eight weeks after contact and CXR was performed 3 months and 12 months after contact.

Active TB was defined as infection with *M. tuberculosis*, with or without symptoms and/or clinical signs, typical CXR and confirmed microbiologically. LTBI was defined as infection with *M. tuberculosis*, without any clinical manifestation, and was diagnosed if TST ≥ 10 mm or IGRA > 0.35 IU/mL after exclusion of active TB regardless of CXR results due to the low sensitivity and specificity of CXR for the diagnosis of LTBI. The prevalence of LTBI when taking up employment (first week of employment) was compared with follow-up examinations.

Only in a subgroup were IGRA and TST performed simultaneously. This is because IGRA testing was introduced two years after the start of the systematic screening. The probability of a positive IGRA based on TST results was calculated for this subgroup. These probabilities were multiplied by the number of HCWs in the whole study group who had the same TST results. Adding up these products gave the number of HCWs that can reasonably be expected to be IGRA-positive in the whole group if they had all been tested with IGRA. This allowed the proportion of IGRA-positive HCWs in the whole study group to be estimated.

Statistical analysis was performed using a two-tailed chi-square test to compare proportions of positive tests (TST and IGRA) and StudentÂ´s t-test, and a simple analysis of variance (one way ANOVA) to compare means and standard deviations between groups. A p-value < 0.05 was considered statistically significant. Analysis was carried out with SPSS, Version 14.

The evaluation was carried out according to the objectives of the Commission for the Prevention and Control of Tuberculosis of Hospital S. João, no additional data was collected, and the analysis was carried out anonymously. For these reasons it was not necessary to request approval by the Ethics Committee.

## Results

The study comprises 5,524 HCWs working or training between September 2005 and June 2009 in our Hospital. TST results are available for 5,209 HCWs (Figure 1). Due to active TB in their medical history, TST was not performed in 234 (4.3%) HCWs. Of these, 33 did not know the year of diagnosis, 88 reported that it had occurred before working as HCW and 113 afterwards. Based on clinical evaluation and CXR, none of these 234 HCWs had active TB at the time of screening.

32.9% of the HCWs had a TST ≥ 15 mm and 55.2% (32.9%+22.3%) had a TST ≥ 10 mm (Table 1). BCG vaccination did not increase the probability of a large TST diameter, with 63.2% of those with a TST < 5 mm compared to 53.9% of those with a TST ≥ 15 mm having a record of BCG vaccination or a vaccination scar. Those with a TST ≥ 15 mm were older and employed for longer time as HCWs than those with a smaller TST diameter (p-value for both trends 0.001). Surprisingly, those with workplaces or tasks assumed to be of low risk most often showed a TST ≥ 15 mm (37% versus 31% for moderate and 33.8% for high risk). On the other hand, they were less likely to have a TST ≤ 5 mm (25.7% versus 34.7% for moderate and 31.7% for high risk) (p-value 0.001). Again surprisingly, physicians were less likely to have a TST ≥ 15 (25.1% versus 38.4% among operational assistants) and more likely to have a TST ≤ 5 mm (41.4% versus 22.6% among administrative assistants, Table 2) (p-value 0.013).

**Table 1:** Risk factors for LTBI by TST

	TST in mm			
	**<5**	**≥5 - <10**	**≥10 - <15**	**≥15**

	**N (%)**	**N (%)**	**N (%)**	**N (%)**

BCG scar or record (%)	1061 (63.2)	446 (68.3)	714 (61.3)	924 (53.9)

Age ± SD (years)	35 ± 10.7	37 ± 11.9	39 ± 12	41 ±10.7

Duration of exposure ± SD	9 ± 9.6	12 ± 11.3	13 ± 11.6	16 ± 10.8

Low Risk (row%)	215 (25.7)	100 (12.0)	212 (25.4)	309 (37.0)

Moderate risk	897 (34.7)	331 (12.8)	558 (21.6)	802 (31.0)

High risk	566 (31.7)	222 (12.4)	394 (22.1)	603 (33.8)

All (%)	1678 (32.2)	653 (12.5)	1164 (22.3)	1714 (32.9)

**Table 2:** Profession by TST

	TST in mm			
	**<5**	**≥5 - <10**	**≥10 - <15**	**≥15**

	**N (%)**	**N (%)**	**N (%)**	**N (%)**

Operational Assistent	234 (35.2)	108 (16.3)	166 (25.0)	256 (38.6)

Administrative Assistent	76 (22.6)	47 (14.0)	84 (25.0)	129 (38.4)

Nurse	533 (28.8)	202 (10.9)	419 (22.7)	695 (37.6)

Physician	528 (41.4)	162 (12.7)	264 (20.7)	320 (25.1)

Technician	163 (35.4)	73 (15.8)	99 (21.5)	126 (27.3)

Others	144 (27.4)	61 (11.6)	132 (25.1)	188 (35.8)

All (%)	1678 (32.2)	653 (12.5)	1164 (22.3)	1714 (32.9)

Since 2005, 53 cases of active TB have been diagnosed (Table 3), of which 19 occurred in 2005, the year the systematic screening started. This was also the year with the highest rate of active TB in the screening population. The number of HCWs with active TB in the population declined in the following years: 13 in 2006, 14 in 2007, 6 in 2008 and 1 HCW with active TB in the first six month of 2009. HCWs considered to be at low risk of TB exposure were less likely to have active TB (0.5%) than those with moderate (1.3%) or high risk of exposure (0.9%) (p-value 0.023). Contrary to the probability of a TST ≥ 10 mm, which was second lowest for physicians among all of the HCWs screened, the probability of active TB was highest for physicians (1.7%) followed by nurses (1.0%) (p-value 0.034).

**Table 3:** Distribution of active TB cases since 2005 (n = 53) according to risk and profession

	TST pos		Active TB		Total
**Risk**	**N**	**%**	**N**	**%**	**N**

Low	521	62.3	4	0.5	836

Moderate	1360	52.6	33	1.3	2588

High	997	55.9	16	0.9	1785

Profession					

Operational Assistent	422	63.6	6	0.9	664

Administrative Assistent	213	63.4	0	--	336

Nurse	1114	60.3	18	1.0	1849

Physician	584	45.8	21	1.7	1274

Technician	225	40.1	4	0.7	561

Others	320	61.0	3	0.6	525

All	2878	55.3	53	1.0	5209

IGRA was performed in 1,686 HCWs (Table 4). For 13 (<1%) HCWs IGRA was indeterminate. This remained the case for 4 of these after the second determination. The subgroup with determinate IGRAs (n = 1,682) was comparable to the whole group (n = 5209) in which TST was performed with respect to gender (female 72% versus 72%), age (36 years Std 10.8 versus 38 years Std 11); and duration of employment in healthcare (11 years Std 10.5 years versus 12 years Std 11, no table).

**Table 4:** IGRA and TST in subgroup simultaneously tested [23]

	IGRA			All		
**TST in mm**	**pos**	**%**	**neg**	**%**	**N**	**Col%**

<5	10	5.3	177	94.7	187	11.1

≥5 - <10	23	12.9	155	87.1	178	10.6

≥10 - <15	168	28.4	423	71.6	591	35.1

≥15	357	49.2	369	50.8	726	43.2

All	558	33.2	1124	66.8	1682	100.0

Out of 1,682 HCWs with a determined IGRA, 558 (33.2%) were positive. Probability of a positive IGRA increased with the diameter of the TST. However, even with a diameter of ≥15 mm only 49.2% of these HCWs had positive IGRA results (Table 4). Applying the probabilities of a positive IGRA for the different diameter category of the TST to all HCWs tested with TST produces a positive IGRA rate of 25.9% (calculated from Table 1 and Table 3).

Comparing the prevalence of LTBI found during the first week of employment (n = 1144) and follow-up examinations (n = 4062), a significantly higher prevalence was found in the latter, with 17.7% versus 29.0% using IGRA and 38.0% versus 60.1% using TST as a criterion for determining LTBI (no table, p-values for both IGRA and TST < 0.001).

## Discussion

Our descriptive data show that the TB burden among Portuguese HCWs is high with 53 out of 5,209 (1%) being diagnosed with active TB in the five-year period from 2005 to 2009. Accordingly, the prevalence of LTBI is high. However, estimates of LTBI prevalence vary to a great extent depending on whether prevalence is assessed with TST or IGRA (55% versus 26%).

Similar variations are found for HCWs in other countries, too. For instance, in a study involving 171 nurses from London the prevalence of LTBI was 16.2% by TST and 7.6% with the IGRA [27]. The relationship between TST and IGRA in this study is similar to the one we report, despite the much higher prevalence of LTBI in our hospital, which probably reflects differences between the two countries regarding the prevalence of LTBI in the general population [7,10,28]. In other studies, the prevalence of LTBI in HCWs has ranged from 22 to 41% with TST (≥10 mm) and of 10 to 40% with IGRA tests [20,27,29,30]. In a review conducted by Menzies *et al *in 2007 [7], the prevalence of LTBI in higher-income countries ranges from 11% to 30% (with TST), while in low-income countries it is estimated between 60% and 80% [21]. In Portugal there are no similar studies for comparison. If we accept the prevalence found in our hospital as representative of the country, Portugal would have a prevalence of LTBI in HCWs that is higher than in countries with high incomes but lower than in low-income countries. In our previous publication, prevalence of LTBI was 33.2% when assessed by IGRA [23]. However, this figure seems to overestimate LTBI prevalence in the total screening population because IGRA was more often performed in HCWs with a higher TST. Prevalence of LTBI is more likely to be in the range of 25% for the whole screening population.

The incidence of *M. tuberculosis *infection in HCWs is related to the incidence in the general population in that geographical area. Added to this is the increased risk as a professional group and work conditions [6-9,31]. In a study by de Vries G *et al *[32], 67 HCWs with TB were evaluated and it was determined that in 42% of these cases the infection had been acquired in the hospital, 28% in the community and 30% abroad.

Another concept for risk assessment examines the relationship between the number of admissions for TB with the number of HCWs. In hospitals with over 200 admissions per year, or a ratio between the number of HCWs and admissions for TB of less than 1/10, the annual risk of infection (ARTI) in HCWs seems to be between 1 and 10% [2]. In the hospital where this study took place, the average number of admissions for TB as the primary diagnosis was 258 per year, giving a ratio of 17.2 HCWs (physicians and nurses) per TB admission and thus ranking it as a moderate-risk hospital [9].

The control of TB as a nosocomial infection requires, above all, the adoption of a "non-reactive" attitude, as it is known that most cases of TB transmission in hospitals occur in places where collective and individual measures of protection were not properly implemented (due to low probability of occurrence) [32]. Therefore, the rapid identification of patients with known or suspected active TB, the rapid implementation of airborne precautions and the use of a surgical mask or N95 respirator by the HCW are necessary measures for active protection. If TB patients are suspected of having MDR/XDR-TB, this might even warrant the use of more effective respirators for the HCWs. Given the low effectiveness of the BCG vaccination [33,34], the strategy for preventing TB should be based on the identification and treatment of LTBI as a way of reducing the number of infected individuals, and the risk of progression to active TB [18].

In our study, the distribution of TB cases was not uniform over the years in question, with a maximum of 19 in 2005 (equivalent to 351/100.000), which is almost eight times higher than the incidence among the general population in the same geographical area (10). Since the implementation of this screening programme, there has been a significant reduction in TB cases. In 2008 only 6 cases of TB were diagnosed and in the first half of 2009 only 1 case. No new measures of infection control were implemented that might explain this effect. We believe that HCWsÂ´ awareness of protective measures increased. They were therefore adhering to the rules more closely. Detection bias might also have a certain influence. At the start of the systematic screening there may have been some cases detected early or cases that would shortly have been detected anyway. In later years this leveled off towards early case detection.

Both TST and IGRA tests have limitations in the diagnosis of LTBI. The main problems with TST depend on technical limitations, difficulty in interpreting the results and the existence of a significant number of false positives [13,35,36]. On the other hand, IGRA tests, despite being more specific and having at least identical sensitivity to TST [20,37-39], present difficulties in interpreting results near the cut-off between positive/negative and also have higher unit costs [24,40]. The absence of a gold standard to correctly identify the sensitivity and specificity of each test poses a challenge [41]. The inability of both tests to distinguish between infection and immunological memory is a further shortcoming. A positive test indicates an immune response to stimulation by mycobacterial antigens, and not necessarily the existence of live *M. tuberculosis *in the human host. The percentage of individuals who are truly infected with *M. tuberculosis *after a TST or IGRA conversion is actually unknown. Therefore the term "latent infection" should be understood as the persistence of immune response and not necessarily as a potential risk for progression to disease [42].

To circumvent the booster effect problem, it is suggested to repeat the TST with a one-week interval (two steps), particularly in populations with high rates of BCG vaccination [13,43-45]. In this study, the difficult interpretation of this effect, the decrease in compliance by repetition of TST and the simultaneous use of IGRA tests were reasons for not performing the two-step TST systematically.

Risk assessment was not confirmed by distribution of TST diameter in our study, e.g. the highest proportion of TST ≥ 15 mm was observed in HCWs assumed to be at low risk of TB exposure. Two effects might explain this seemingly contradictory observation. First, risk classifications are based on a certain stability of professionals in the workplace [9], which generally is not observed. Second the habits, training and awareness necessary for taking personal protection measures, and socioeconomic characteristics of each group can confound the association between positive TST and risk assessment. Analysing the rate of active TB rather than positive TST gave a better association between perceived risk and actual TB burden, e.g. physician and nurses had the highest rates of active TB and those with low risk had the lowest rate of active TB.

Concerning limitations of the study, selection bias is of major concern. There is a certain selection bias because HCWs with recent contact with TB patients and HCWs with high TST diameters in their medical history are more likely to have screening performed. This explains why IGRA positivity is higher in the subgroup with simultaneous TST and IGRA testing than in the whole group. Therefore the rate of positive IGRAs estimated for the whole group is more likely to be the proportion of positive IGRA results (25.9%) to be expected for HCWs in comparable hospitals in Portugal.

## Conclusions

The TB burden in Portuguese HCWs working in comparable hospitals is high. The screening of these professionals is essential for an early diagnosis of active disease. It is also essential to identify cases with higher risk of future progression to disease as these professionals are most likely to benefit from preventive chemotherapy.

Since the implementation of this screening programme, the incidence of TB has decreased, which supports the importance of TB screening as a disease control measure, both by identifying high-risk cases and by alerting HCWs to this problem.

The authors declare that they do not have any direct or indirect personal relationship, affiliation or association with any party with whom they deal in their day to day work that would give rise to any actual or perceived conflict of interest.

## Authors' contributions

JTC designed the study, performed the physical examinations, took part in data analyse and wrote the manuscript. RS was involved in data collection and analysis, and drafting of the paper. MJC was involved in designing the study and data collection, and gave substantial critical comments for manuscript writing. AN was involved in data analysis and gave substantial critical comments for manuscript writing. All authors have read and approved the final manuscript.
